# Pediatric Medial Discoid Meniscus: Case Series and Postoperative Outcomes

**DOI:** 10.3390/children12050646

**Published:** 2025-05-16

**Authors:** Franck Accadbled, Oliwer Sygacz, Joe Rassi, Alexandru Herdea

**Affiliations:** 1 Department of Orthopedics, Children’s Hospital, Centre Hospitalier Universitaire de Toulouse, 31300 Toulouse, France; accadbled.f@chu-toulouse.fr (F.A.); rassi.j@chu-toulouse.fr (J.R.); 2 Department of Paediatric Orthopaedics and Rehabilitation, Medical University, 20-093 Lublin, Poland; 52234@student.umlub.pl; 3 11th Department of Pediatric Orthopedics, “Carol Davila” University of Medicine and Pharmacy, Bd. Eroii Sanitari Nr. 8, 050474 Bucharest, Romania; 4 Pediatric Orthopedics Department, “Grigore Alexandrescu” Children’s Emergency Hospital, 011743 Bucharest, Romania

**Keywords:** discoid meniscus, medial meniscus, meniscal repair, meniscal saucerization

## Abstract

Discoid medial meniscus (DMM) is a rare congenital anomaly, with bilateral cases being even more uncommon. Due to its rarity, comprehensive data on its prevalence, clinical presentation, and optimal management strategies are limited. This study aimed to evaluate the prevalence, clinical characteristics, and treatment outcomes of symptomatic DMM in a pediatric population. A retrospective review was conducted on patients under 18 years of age diagnosed with symptomatic DMM. Data were extracted using ICD-10 codes Q68.6 and M23.16 and were supplemented by free-text searches. The inclusion criteria encompassed a confirmed DMM diagnosis, availability of MRI and radiographic imaging, complete clinical documentation, and a minimum of six months of postoperative follow-up. Demographic data, clinical presentations, imaging findings, surgical interventions, and outcomes were analyzed. Three patients (five knees) met the inclusion criteria. All presented with symptomatic DMM requiring surgical intervention. Arthroscopic saucerization combined with meniscal repair was performed in all cases. Postoperative follow-up revealed that two of the patients achieved excellent outcomes, while one required three reoperations due to retearing but remained symptom-free at one year post-revision. Radiographic assessments did not reveal characteristic changes typically associated with DMM. Symptomatic DMM, though rare, may be encountered more frequently than previously reported, especially when focusing on symptomatic cases. Arthroscopic saucerization with concurrent meniscal repair appears to be an effective treatment modality, yielding favorable mid-term outcomes. Given the limited number of cases and the variability in their presentations, further research with larger cohorts is essential to establish standardized management protocols and to better understand the long-term prognosis of patients with this rare condition.

## 1. Introduction

Discoid meniscus is the most common congenital anomaly of the knee. It is characterized by an abnormally thickened and enlarged meniscal morphology, typically affecting the lateral meniscus. Discoid lateral meniscus is relatively uncommon, with prevalence rates estimated between 1.2% and 5.2%, whereas involvement of the medial meniscus is exceedingly rare, with rates of only 0.12% to 0.3% [1,2]. Discoid meniscus is frequently asymptomatic and may go undiagnosed without imaging or surgical intervention. Epidemiological studies suggest a significantly higher prevalence in Asian populations, with reported rates of up to 13% in Japan and 10.6% in Korea, compared to 3–5% in Western countries. Despite its relatively high occurrence, particularly in certain ethnic groups, its true prevalence remains uncertain due to its often-silent clinical presentation [3,4,5]. Bilateral knee involvement in discoid meniscus cases has been reported in 79% to 97% of patients [6].

The etiology of discoid meniscus (DM) remains a subject of debate, with ongoing discussion as to whether it represents a true congenital malformation or a variation in normal meniscal development. Observations from fetal cadaveric studies indicate that a discoid configuration can be present during early gestation, with progressive remodeling typically occurring between the second and third trimesters. However, in some cases, this developmental transition may be incomplete, resulting in the persistence of the discoid morphology postnatally. The underlying mechanisms responsible for this persistence remain poorly understood [4,7,8].

The clinical presentation of discoid meniscus varies depending on factors such as age, meniscal stability, and concurrent intra-articular pathology. Commonly reported symptoms include joint snapping, episodic pain, swelling, and mechanical locking. An audible “click” may be noted during the final degrees of knee extension [6]. Due to the typically vague and intermittent nature of the symptoms, along with the variable reliability of physical examination findings, advanced imaging—particularly MRI—is often essential for accurate diagnosis and treatment planning. The initial imaging approach typically includes standard knee radiographs comprising anteroposterior, lateral, tunnel, and skyline projections [6,9,10,11].

Surgical management of discoid meniscus is typically reserved for cases presenting with symptomatic tears, instability, or ongoing pain, with or without mechanical issues such as flexion contracture in pediatric patients. Asymptomatic discoid meniscus does not require operative treatment; however, patients should be informed about the potential risk of future injuries, especially if this condition is identified incidentally or found in the opposite knee [12].

In this case series, the aims were to evaluate the prevalence of medial discoid meniscus in the examined population; to identify its distinguishing morphological and imaging characteristics; and to outline current strategies and recommendations for the diagnosis, treatment, and management of patients presenting with this rare anatomical variant.

## 2. Materials and Methods

### 2.1. Study Design

This retrospective study reviewed medical records of pediatric patients who underwent surgery for medial discoid meniscus at the Centre Hospitalier Universitaire (CHU)–Hôpital des Enfants, a tertiary urban pediatric hospital in Toulouse, France. The study period spanned from December 2005 to December 2024. Ethical approval was granted by the CHU–Hôpital des Enfants Ethics Committee under approval number RnIPH 2025-66.

### 2.2. Participants

Data were extracted from the electronic medical records stored in the internal system of CHU–Hôpital des Enfants using the ICD-10 diagnostic codes Q68.6 (discoid meniscus) and M23.16 (discoid meniscus, congenital), as well as through a free-text search. Out of 754 meniscal procedures, a total of 70 discoid meniscus cases were identified. Three patients with medial discoid meniscus were selected for inclusion.

Demographic data such as age, gender, and knee laterality (right or left) were recorded. Clinical information including the final diagnosis, patient history, physical examination findings, and paraclinical tests was also collected and analyzed.

The inclusion criteria for this study were as follows: patients aged below 18 years diagnosed with medial discoid meniscus; availability of unilateral or bilateral knee MRI, AP and lateral knee X-rays, complete clinical examination and patient history, and surgical records; and a minimum postoperative follow-up of six months.

### 2.3. Outcome Measures

This study aimed to determine the prevalence of medial discoid meniscus among the pediatric patients in our case series and to evaluate clinical outcomes following arthroscopic treatment, with a particular focus on returning to sports, recurrence of symptoms, and clinical assessment of treatment efficacy.

## 3. Results

This study presents three pediatric cases of medial discoid meniscus, involving five knees in total, that were managed arthroscopically and followed postoperatively. A patient summary is shown in Table 1.

### 3.1. Case 1—P.E.

**Figure 1:**
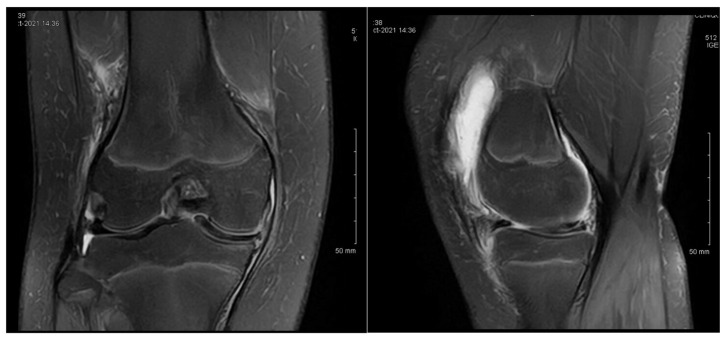
Patient P.E. (12 years old). MRI images of the right knee demonstrating features of a medial discoid meniscus (**left**—coronal view; **right**—sagittal view). The coronal T2-weighted image showed a thickened and abnormally widened medial meniscus extending across the medial tibial plateau, consistent with a discoid morphology. The sagittal view revealed increased signal intensity and an altered morphology in the posterior horn. Mild joint effusion was also present. An initial MRI was performed before the first surgery.

P.E., a 12-year-old female, was diagnosed with bilateral medial discoid meniscus, although she was asymptomatic on the left side. At presentation, she reported chronic medial knee pain dating back several years, with clinical findings of joint effusion and a normal range of motion. MRI images can be seen in Figure 1. She was not engaged in any sporting activities.

**Figure 2:**
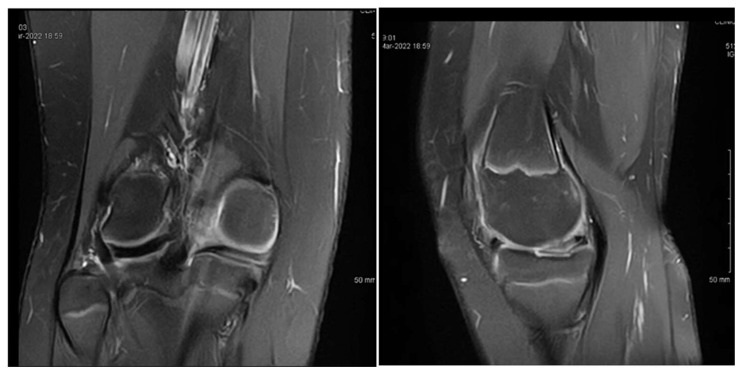
Patient P.E. (12 years old). Follow-up MRI images of the right knee after an initial arthroscopic partial meniscectomy in a pediatric patient with medial discoid meniscus (**left**—coronal T2 view; **right**—sagittal T2 view). The coronal image revealed persistent thickening and an abnormal morphology in the medial meniscus, consistent with a residual discoid shape. The sagittal view demonstrated an altered meniscal contour with persistent signal heterogeneity in the posterior horn, suggesting incomplete healing or retearing. Mild joint effusion was still present, indicating ongoing intra-articular inflammation.

At the age of 12, she underwent arthroscopic exploration, which revealed a complete medial discoid meniscus in the symptomatic knee. This was treated with a partial meniscectomy. At her 6-month follow-up, she continued to experience knee pain and persistent, non-puncturable effusion. A follow-up MRI was performed, and the images can be seen in Figure 2.

**Figure 3:**
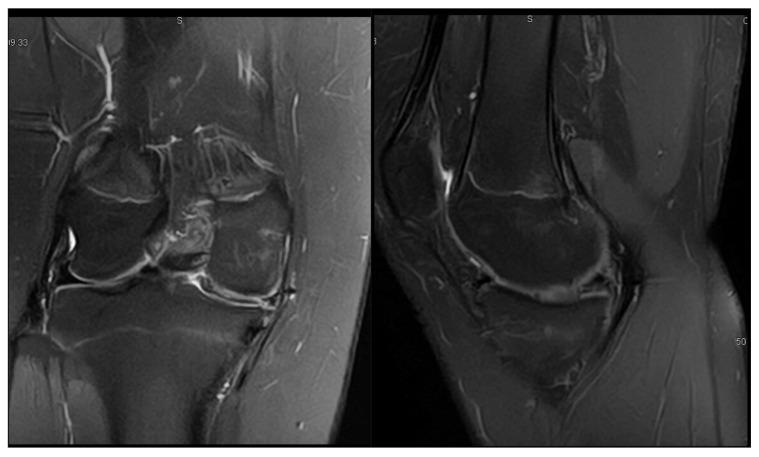
Patient P.E. (13 years old). One-year postoperative MRI of the right knee following the second arthroscopic intervention for medial discoid meniscus (**left**—coronal T2 view; **right**—sagittal T2 view). The coronal image showed a residual discoid configuration in the medial meniscus with persistent abnormal signal intensity. The sagittal slice revealed a lack of clear meniscal healing in the posterior horn, consistent with incomplete healing or suture failure. Mild effusion was still present. Clinically, the patient reported ongoing pain and activity limitations at this stage.

A second arthroscopic procedure was performed at age 13, during which a posterior horizontal meniscal tear was identified and treated with meniscal suturing. However, the 1-year follow-up revealed persistent pain, localized tenderness in the medial compartment, a small effusion, and a need to restrict sports activities. The MRI findings at that time showed no evidence of meniscal healing, as seen in Figure 3.

At the age of 14, a third arthroscopic intervention was performed, during which a radial tear was identified and treated with additional meniscal suturing. Her 1-year follow-up after the third surgery showed a favorable evolution. She had resumed physical activities at school without complaints, and no further effusion or pain was reported.

### 3.2. Case 2—S.C.S.

**Figure 4:**
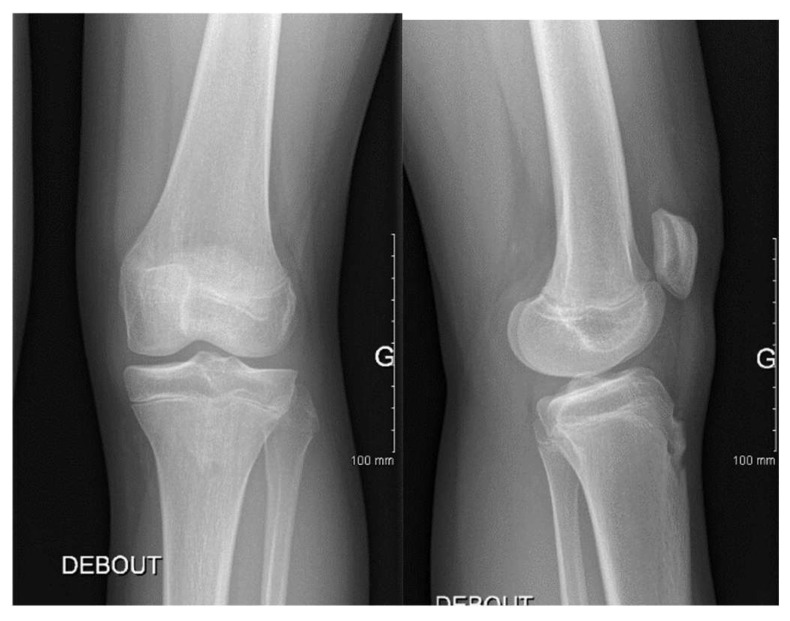
Patient S.C.S. (13 years old). Anteroposterior and lateral knee radiographs of a patient diagnosed with medial discoid meniscus. The standing anteroposterior (**left**) and lateral (**right**) X-ray views showed no obvious bony abnormalities. The joint space appeared to be preserved, with no signs of degenerative changes or malalignment.

**Figure 5:**
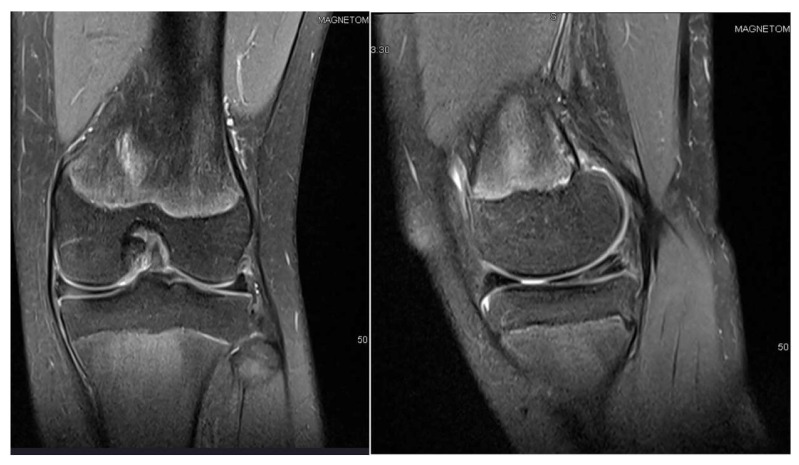
Patient S.C.S. (13 years old). Preoperative MRI of the left knee diagnosed with a medial discoid meniscus (**left**—coronal T2 view; **right**—sagittal T2 view). The coronal image demonstrated an abnormally enlarged and thickened medial meniscus extending across the tibial plateau, consistent with a complete discoid morphology. The sagittal view revealed a horizontal cleavage tear in the posterior horn.

S.C.S., a 13-year-old female dancer, presented with left knee pain and occasional mechanical locking. Clinical examination revealed a normal range of motion without effusion. The symptoms had started approximately six months prior to the consultation. An X-ray is shown in Figure 4, and the corresponding MRI can be seen in Figure 5.

This patient underwent arthroscopic surgery at the age of 13. During surgery, a horizontal tear in the posterior horn of the medial discoid meniscus was found and treated with a partial meniscectomy and a meniscal suture.

**Figure 6:**
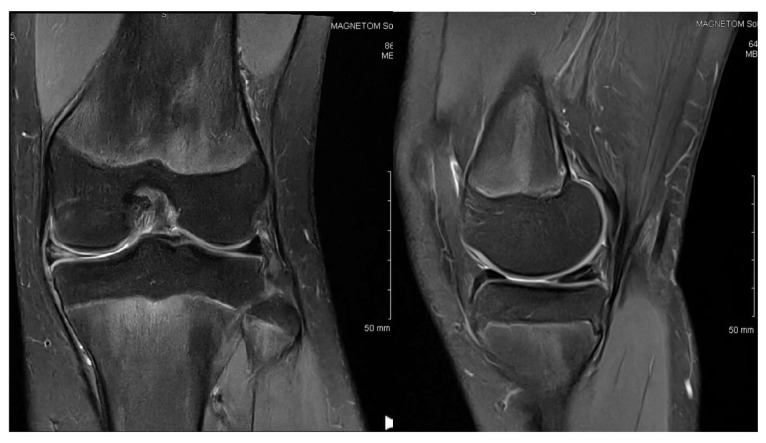
Patient S.C.S. (13 years old). Six-month postoperative MRI of the left knee following arthroscopic treatment for a medial discoid meniscus (**left**—coronal T2 view; **right**—sagittal T2 view). The coronal view showed normalization of the meniscal contour compared to the preoperative images, with resolution of the meniscal thickening. The sagittal view demonstrated no signs of retearing or signal changes in the posterior horn. No joint effusion was observed. These imaging findings were consistent with her clinical improvement and successful return to activity.

At her 3-month follow-up, the patient was asymptomatic, with a full range of motion and no effusion. She had resumed dancing without limitations. These results were maintained at her 6-month follow-up, with no recurrence of symptoms. A follow-up MRI was performed, and images can be seen in Figure 6.

### 3.3. Case 3—G.L.

**Figure 7:**
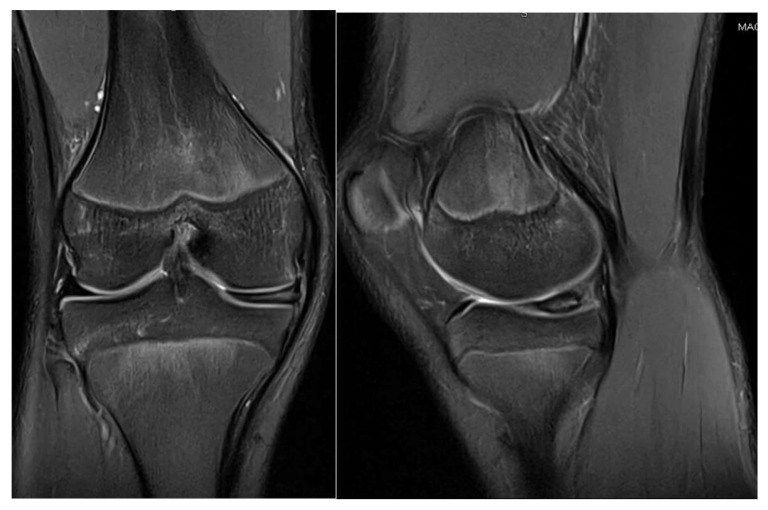
Patient G.L. (14 years old). Preoperative MRI of the right knee with symptomatic medial discoid meniscus (**left**—coronal T2 view; **right**—sagittal T2 view). The coronal view demonstrated a widened and abnormally thickened medial meniscus extending into the intercondylar notch, characteristic of a discoid morphology. The sagittal view showed a horizontal signal cleft in the posterior horn, consistent with a horizontal tear. These findings led to an arthroscopic partial meniscectomy and meniscal suturing.

**Figure 8:**
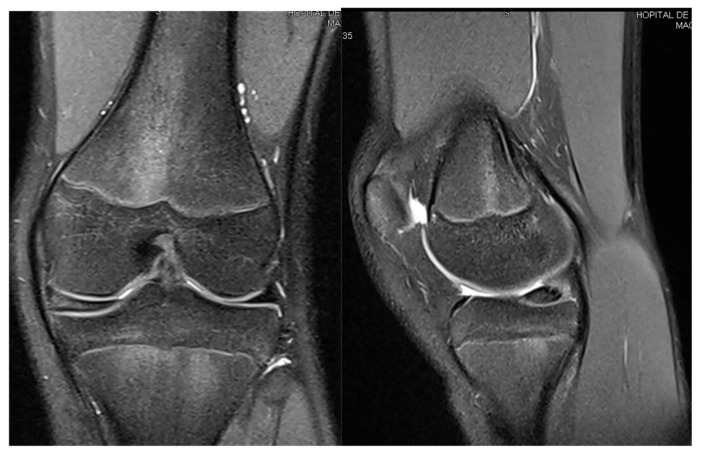
Patient G.L. (14 years old). MRI of the left knee in a patient with bilateral symptoms of medial discoid meniscus (**left**—coronal T2 view; **right**—sagittal T2 view). The coronal view revealed a widened medial meniscus with an abnormal bow-tie appearance spanning more than two contiguous slices, indicative of a discoid morphology. The sagittal image showed increased signal intensity within the posterior horn, suggesting possible early tearing or degeneration. These findings supported bilateral involvement, although only the right knee underwent surgical treatment during the study period.

G.L., a 14-year-old male basketball player, presented with bilateral knee pain that was more pronounced medially. On examination, he had a full range of motion and medial joint line tenderness, without joint effusion. Right knee MRI images are shown in Figure 7, while left knee MRI images are presented in Figure 8.

**Figure 9:**
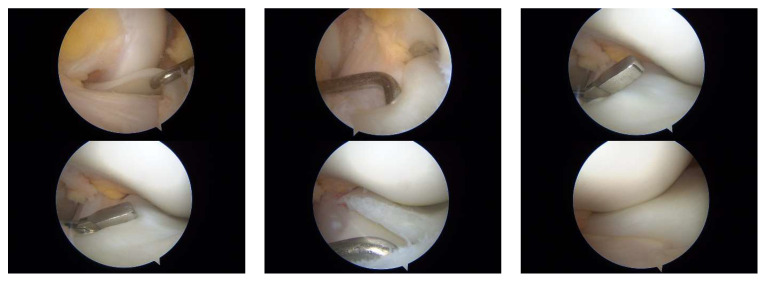
Patient G.L. (14 years old). Intraoperative arthroscopic views of the medial compartment of the knee in a pediatric patient with medial discoid meniscus. Sequential arthroscopic images demonstrate the surgical findings and treatment process. (**Top row**): Visualization of the abnormally thickened medial discoid meniscus. The probe demonstrates the loss of the normal meniscal contour and mobility. (**Middle lower row**): Meniscal rasping and preparation of the tear site prior to suturing. The posterior horn and central segment are accessed using arthroscopic tools. (**Bottom row**): Placement of all-inside sutures to stabilize the tear and reshape the meniscus. Final inspection shows an improved morphology with a restored contour and stable fixation.

At age 14, he underwent arthroscopic surgery on the more symptomatic knee (the right knee). An incomplete medial discoid meniscus with a horizontal tear in the posterior horn was identified and treated with a partial meniscectomy and meniscal suturing. At the 6-month follow-up, he reported no pain or effusion and had regained a full range of motion. Intraoperative images are shown in Figure 9, and Supplementary Video S1 provides a dynamic assessment of the medial discoid meniscus.

## 4. Discussion

The concept of a discoid meniscus was first introduced in 1889, when Young et al. [13] identified an abnormal, disc-shaped meniscus in the lateral compartment of the knee. It was not until 1941 that Cave [14] expanded this understanding by demonstrating that a similar discoid configuration could also occur on the medial side of the knee joint. While lateral discoid menisci are observed in approximately 1.2% to 5.2% of the population, medial discoid menisci are exceedingly rare, with reported prevalence rates ranging from 0.06% to 0.3% [15]. Reports of bilateral medial discoid menisci are scarce in the literature, and determining their true prevalence is challenging, as a significant number of discoid menisci may remain asymptomatic and thus go undiagnosed [16]. In our study population, symptomatic medial discoid menisci were identified with a higher frequency than typically reported. Only cases presenting with clinical symptoms were considered in the analysis.

Although the literature contains studies on medial discoid menisci, the majority are case reports. These reports, similar to our study, describe both bilateral and unilateral occurrences of medial discoid menisci [17,18,19]. In our case series, statistical analysis was limited due to the small number of participants.

The clinical manifestations of a torn medial discoid meniscus closely resemble those of a standard meniscal tear, typically presenting with pain, swelling, and mechanical symptoms such as locking or catching. In contrast, lateral discoid meniscus tears may exhibit a distinctive ‘snapping’ sensation during knee movement, a feature less commonly associated with medial discoid meniscus injuries [20,21]. In our case series, the patients exhibited symptoms consistent with those typically associated with medial discoid meniscus tears. Notably, despite the presence of meniscal damage, all individuals maintained a full range of knee motion.

The MRI diagnostic criteria for a discoid medial meniscus mirror those established for the lateral variant. On sagittal images, a discoid meniscus is suggested when the anterior and posterior horns appear continuous across three or more consecutive slices, each 5 mm thick, forming a characteristic ‘bow tie’ sign. In the coronal plane, a discoid meniscus is indicated when the ratio of the minimum meniscal width to the maximum tibial width exceeds 20% [22].

Radiographic assessment of the knee joint typically reveals normal bony structures; however, in cases of discoid medial meniscus, extension of the meniscal edge into the femoral intercondylar region can lead to widening of the medial joint space. Characteristic radiographic features include a medial tibial plateau with a ‘cup-like’ appearance, collapse of the proximal medial joint, flattening of the femoral condyle, and an enlarged medial joint space [23]. In our study, such radiographic changes were not observed in any of the evaluated cases.

In a study conducted by Feroe et al., out of 446 knees examined, 12 (2.7%) were identified with a discoid meniscus. Among them, meniscal tears were present in nine cases (75%), predominantly exhibiting horizontal cleavage patterns. Surgical intervention involved saucerization in 11 knees (92%), and in 7 cases (58%), medial meniscal repair was performed when deemed necessary [24]. The literature documents cases of medial discoid menisci managed with saucerization alone, as well as those requiring additional meniscal repair. For instance, Desai et al. reported a case involving an 18-year-old male with a symptomatic tear in the posterior horn of a discoid medial meniscus that was treated successfully with arthroscopic saucerization and repair [25]. Conversely, Al Saedi et al. described a 13-year-old male patient who underwent saucerization alone for a discoid medial meniscus tear, resulting in a favorable outcome without the need for repair [26].

Due to the rarity of discoid medial meniscus cases, comprehensive treatment guidelines are lacking. Most of the available literature consists of individual case reports or small series, making it challenging to establish standardized management protocols. Additionally, regenerative and biological therapies, such as platelet-rich plasma, have been explored as adjunct treatments to enhance healing in various sports-related injuries, particularly among young athletes [27].

Two limitations of our study are its retrospective nature and its small sample size, which restricted our ability to perform meaningful statistical analysis. As a result, the generalizability of our findings is limited, and conclusions should be interpreted with caution.

In our case series, two out of three patients (66%) achieved excellent outcomes following surgical interventions for symptomatic medial discoid meniscus tears. One patient required reoperation; however, after three years of follow-up, they remained symptom-free. These results align with the existing literature. For instance, Feroe et al. reported a retear rate of 36% (four out of eleven knees) at an average of 25.8 months postoperatively, with two knees necessitating revision surgery [24]. While many authors report favorable outcomes for medial discoid meniscus treatments [15,28,29], the rarity of this condition limits the size of study cohorts, making it challenging to draw definitive conclusions. Further research with larger sample sizes is needed to establish standardized treatment protocols and long-term prognoses.

## 5. Conclusions

DMM is an exceptionally rare anatomical anomaly, with bilateral cases being even more uncommon. Prompt MRI of the knee is instrumental in preoperative identification of discoid meniscus anomalies, facilitating accurate diagnosis and surgical planning. Arthroscopy remains the definitive method for both diagnosing and managing discoid menisci. Given the limited number of cases and the variability in their presentations and treatment approaches, establishing standardized management protocols for DMM remains challenging.

## Figures and Tables

**Table 1:** Summary of pediatric medial discoid meniscus cases, including age at diagnosis, surgical history with corresponding ages, and follow-up outcomes.

Patient	Age at Diagnosis	Knee(s) Involved	Initial Symptoms	Sports Activity	Surgical History	Follow-Up Outcome
P.E.	12	Bilateral (symptomatic right)	Chronic pain, effusion, normal ROM	None	3 surgeries: partial meniscectomy (12 years), suture for horizontal tear (13 years), suture for radial tear (14 years)	1 year after third surgery: symptom-free, resumed school sports
S.C.S.	13	Left	Pain, occasional locking, normal ROM	Dancing	1 surgery: partial meniscectomy and suture for horizontal tear (13)	6 months: symptom-free, full ROM, resumed dancing
G.L.	14	Bilateral (symptomatic right)	Pain, medial tenderness, normal ROM	Basketball	1 surgery: partial meniscectomy and suture for horizontal tear (14)	6 months: symptom-free, full ROM

## Data Availability

The original contributions presented in this study are included in this article and the Supplementary Materials. Further inquiries can be directed to the corresponding author.

## Supplementary Materials

The following supporting information can be downloaded at https://www.mdpi.com/article/10.3390/children12050646/s1, Supplementary Video S1: Dynamic arthroscopic exploration of the medial discoid meniscus. This video highlights the intraoperative assessment of mobility; the tear configuration; and the meniscal repair technique, including debridement and suture passage.
